# Optical Biosensors for Diagnostics of Infectious Viral Disease: A Recent Update

**DOI:** 10.3390/diagnostics11112083

**Published:** 2021-11-10

**Authors:** Atul Sharma, Rupesh Kumar Mishra, K. Yugender Goud, Mona A. Mohamed, Shekher Kummari, Swapnil Tiwari, Zhanhong Li, Roger Narayan, Lia A. Stanciu, Jean Louis Marty

**Affiliations:** 1 Department of Pharmaceutical Chemistry, SGT College of Pharmacy, SGT University, Budhera, Gurugram 122505, Haryana, India; atul_fphs@sgtuniversity.org; 2 Bindley Bio-Science Center, Lab 222, 1203 W. State St., Purdue University, West Lafayette, IN 47907, USA; 3 School of Materials Engineering, Purdue University, 701 West Stadium Avenue, West Lafayette, IN 47907, USA; 4 Department of NanoEngineering, University of California San Diego, La Jolla, CA 92093, USA; 5 Pharmaceutical Chemistry Department, National Organization for Drug Control and Research (NODCAR), Egyptian Drug Authority, Giza 99999, Egypt; mona7722@aucegypt.edu; 6 Department of Chemistry, National Institute of Technology, Warangal 506004, Telangana, India; shekharkummari@nitw.ac.in; 7 School of Studies in Chemistry, Pt. Ravishankar Shukla University, Raipur 492010, Chattisgarh, India; swapnil.tiwari7@gmail.com; 8 School of Medical Instrument and Food Engineering, University of Shanghai for Science and Technology, 516 Jungong Road, Yangpu District, Shanghai 200093, China; zhli@sspu.edu.cn; 9 Department of Materials Science and Engineering, NC State University, Raleigh, NC 27695, USA; rjnaraya@ncsu.edu; 10 Joint Department of Biomedical Engineering, North Carolina State University, Raleigh, NC 27695, USA; 11 BAE-LBBM Laboratory, University of Perpignan via Domitia, 52 Avenue Paul Alduy, CEDEX 9, 66860 Perpignan, France

**Keywords:** viral disease, optical biosensors, diagnostics, nanomaterial, fluorescence, SARS-CoV-2

## Abstract

The design and development of biosensors, analytical devices used to detect various analytes in different matrices, has emerged. Biosensors indicate a biorecognition element with a physicochemical analyzer or detector, i.e., a transducer. In the present scenario, various types of biosensors have been deployed in healthcare and clinical research, for instance, biosensors for blood glucose monitoring. Pathogenic microbes are contributing mediators of numerous infectious diseases that are becoming extremely serious worldwide. The recent outbreak of COVID-19 is one of the most recent examples of such communal and deadly diseases. In efforts to work towards the efficacious treatment of pathogenic viral contagions, a fast and precise detection method is of the utmost importance in biomedical and healthcare sectors for early diagnostics and timely countermeasures. Among various available sensor systems, optical biosensors offer easy-to-use, fast, portable, handy, multiplexed, direct, real-time, and inexpensive diagnosis with the added advantages of specificity and sensitivity. Many progressive concepts and extremely multidisciplinary approaches, including microelectronics, microelectromechanical systems (MEMSs), nanotechnologies, molecular biology, and biotechnology with chemistry, are used to operate optical biosensors. A portable and handheld optical biosensing device would provide fast and reliable results for the identification and quantitation of pathogenic virus particles in each sample. In the modern day, the integration of intelligent nanomaterials in the developed devices provides much more sensitive and highly advanced sensors that may produce the results in no time and eventually help clinicians and doctors enormously. This review accentuates the existing challenges engaged in converting laboratory research to real-world device applications and optical diagnostics methods for virus infections. The review’s background and progress are expected to be insightful to the researchers in the sensor field and facilitate the design and fabrication of optical sensors for life-threatening viruses with broader applicability to any desired pathogens.

## 1. Introduction

A virus is a microscopic parasite and is the most minor transmittable agent, usually much smaller than bacteria. Viruses lack the capacity to survive and replicate outside of a host body [1,2]. They have been the cause of several diseases, such as Japanese encephalitis, Chikungunya, dengue, Ebola, influenza, hepatitis, flu, chicken pox, AIDS, severe acute respiratory syndrome (SARS), and several others [3,4]. A transportable viral particle comprises nucleic acids and an external shell of proteins. The most well-studied viruses have either RNA or DNA as genetic material to encrypt proteins [5]. These viruses are capable of fast replication and distribution and cause diseases, and hence, become a severe threat to human health. Viruses enter the host body and use their machinery for metabolism and self-replication [6]. Viruses have the ability to transmute quickly, along with a complicated exchange amid diverse aspects such as the universal movement of animals/humans and environmental factors contributing to the development of transmissible diseases [7]. Therefore, a specific analytical method is paramount for early virus screening and analysis [8]. A recent example of viral infection is the COVID-19 pandemic, which has already caused the death of more than 3.5 million people worldwide [9,10]. In the literature, various methods for detecting and identifying viruses have been documented, including microbiological and biochemical testing, genetic engineering methods, and immunological procedures [10,11].

Conventional viral detection methods include an enzyme-based antibody assay and a polymerase chain reaction (PCR)based qualitative assay, viral separation, and immunofluorescence based on microscopy, which is not suitable for repetitive clinical testing [12,13]. These techniques necessitate a long turnaround time (2 to 14 days), which is inadequate for combatting rapid community spread. The costs of current viral detection tests are also high. Therefore, rapid, reliable, and reproducible diagnostic methods are vital and “the need of the hour” to recognize pathogenic agents in patients’ biological fluids [14]. Biosensors hold considerable promise as an alternate analytical tool to the existing methods for viral detection, which can help provide timely detection and intervention [15,16].

Optical biosensors have been developed for many years in diverse fields. Over the past decade, movement within the arena of optical biosensor development has been fast-paced, and numerous optical biosensing platforms have been explored for sensitive and label-free detection; these include, but are not limited to, surface plasmon resonance [17,18,19], interferometers [20], ring-resonators [21], photonic crystals [22,23,24], fiber-optics [17,25], and planar optical waveguides [26,27]. The advantages of optical sensors include immunity to electromagnetic interference, remote sensing ability, miniaturization of the assays, intrinsic safety, and the ability to offer multiplexed recognition within a single device [28]. One of the most substantial downsides of regularly used optical sensing methods is the permeation depth of the evanescent field, which is usually less significant than the average size of a bacterial cell, stemming from the incapability to sense higher particulate antigens with sufficient sensitivity [29]. Countless of the current techniques also involve expensive instrumentation to convey the signal read-out. In turn, optical biosensors have been developed to investigate numerous analytes such as proteins, nucleic acid, bacteria, biomarkers, and environmental contaminants, to only name a few [30,31,32,33,34,35]. Optical biosensors are known to be extremely sensitive and can offer ultra-low detection levels, a linear output, low-power consumption, and high resolution [36,37]. Furthermore, they offer excellent repeatability, accuracy, and the ability to be miniaturized. In this work, we will present an overview of viral infectious diseases and their biomarkers, a discussion of recent literature on optical biosensors for viral diagnostics, and a summary of the current state of the art of the field.

## 2. Viral Infectious Diseases

### 2.1. COVID-19

Coronaviruses are a large class of viruses, and their occurrences are common in people [38]. They also known to cause various illnesses, from the common cold to severe acute respiratory syndrome (SARS) [39]. The severe acute respiratory syndrome coronavirus 2 (SARS-CoV-2) causes COVID-19, as it is now called, and is rapidly spreading from its origin in Wuhan City of the Hubei Province in China to the rest of the world [16]. Globally, approximately 167.5 million cases of COVID-19 and approximately 3.5 million deaths have been reported as of 25 May 2021 [9]. For instance, about 27.16 million confirmed cases and 0.31 million deaths have been reported in India [9]. The WHO announced COVID-19 to be a pandemic disease on 11 March 2020 [40,41]. Initially, during the Covid-19 outbreak no effective treatment strategy was formulated; hence, countries were dependent on social distancing, wearing masks, and lockdowns [42,43]. Fever, dry cough, weariness, and muscle soreness are among the symptoms of this condition, which also includes headache, lymphopenia, and dyspnea. Some people also experience nausea, constipation, and diarrhea-like conditions after 2–3 days [16,44]. Elderly people and those who have chronic diseases such as asthma, diabetes, or hypertension seem to be at higher risk for developing severe complications from COVID-19 infection [45], while the disease has a mild effect in children [46,47].

The biosensor is a capable diagnostic tool that appears to be an alternative to many existing analytical methods. Recently, an optical microfluidic chip-based biosensor was developed to detect antibodies against SARS-CoV-2 spike protein [48]. The sensing mechanism relies on the localized surface plasmon resonance (LSPR) involving gold nanospikes (fabricated by electrodeposition) in a microfluidic device coupled with an optical probe. The developed sensing platform achieved a detection level down to 0.5 pM in 30 min. The diagnostic platform showed great potential to supplement current serological assays and improve COVID-19 diagnosis. In other reported work, Ashab Uddin et al.; proposed a surface plasmon resonance (SPR) structure based on the Kretschmann configuration incorporating layers of silicon and BaTiO_3_ on top of Ag for real-time detection of SARS-CoV-2 using thiol-tethered DNA as a ligand [49]. This study performed an extensive numerical analysis based on transfer matrix theory. About a 7.6 times enhanced sensitivity was obtained using the proposed architecture for SARS-CoV-2 detection compared to the basic Kretschmann configuration. Consequently, the proposed sensor design provided an appropriate pattern for highly sensitive, swift, and noninvasive biosensing, which could be beneficial if implemented in experimental sensing protocols.

Meanwhile, Murugan et al. (2020) described a plasmonic fiber-optic (P-FAB) platform for one-step, wash-free detection of SARS-CoV-2 virus particles in saliva samples with minimal sample pre-processing [50]. The P-FAB, which is based on a U-bent optical fiber sensor system, is a handy and sensitive diagnostic platform that has been used to detect a variety of biomolecular analytes for a long time. The two plasmonic, labeled and label-free immunoassays suggested here on the susceptible P-FAB platform could be a good option for diagnosing 10^6^ particles/mL COVID-19 in 15 min.

### 2.2. Middle East Respiratory Syndrome (MERS)

MERS is a viral respiratory disease caused by a novel coronavirus (Middle East respiratory syndrome coronavirus, or MERS-CoV) initially detected in Saudi Arabia in 2012 [51,52]. Fever, cough, and shortness of breath are common MERS symptoms. Pneumonia is a frequent ailment; however, it is not always present [51]. Symptoms of the gastrointestinal tract, such as diarrhoea, have also been observed. Most of these asymptomatic infections were discovered after a thorough contact tracing of a laboratory-confirmed case. However, some laboratory-confirmed MERS-CoV infections are described as asymptomatic, which means they have no clinical symptoms [53]. Despite this, a laboratory test revealed that the cause of death for 35% of patients who have died was MERS-CoV [54]. In the literature, the development of several biosensors have been written for MERS detection [55,56,57]. For instance, MERS-CoV was specifically detected using a label-free colorimetric assay by employing gold nanoparticles. Colorimetric tests are a representative tool used to easily classify the target analyte in the matrix via color changes of an indicator.

Kim et al. proposed a colorimetric test relying on an expanded form of double-stranded DNA (dsDNA) self-assembly shielded gold nanoparticles (AuNPs) under an electrolyte-rich medium to detect MERS-CoV [58]. A localized surface Plasmon resonance shift and color variations of AuNPs in the UV–vis wavelength range could be used to validate the presence of viral molecules on this platform. The authors created a pair of thiol-modified probes with complementary base pairs upstream of the E protein gene (upE) and open reading frames (ORF) 1a on MERS-CoV at either the 5′ or 3′ end. The disulfide-induced extended self-assembled complex formed by the target and probes’ dsDNA shields AuNPs against salt-induced aggregation and optical property transition. This colorimetric assay was able to distinguish 30 bp MERS-CoV at 1.0 pmol L^−1^. In another approach, a multiplexed paper-based colorimetric DNA sensor was developed using pyrrolidinyl peptide nucleic acid-induced AgNPs aggregation for the detection of MERS-CoV [59]. By monitoring the color change of AgNPs, the oligonucleotide target was found, with detection limits of 1.53 nM for MERS-CoV. The acpcPNA probe demonstrated significant selectivity for complementary oligonucleotides over single-base mismatch, two-base mismatch, and noncomplementary DNA targets in this research.

### 2.3. Human Immunodeficiency Virus (HIV)

Around 1920, the human immunodeficiency virus arrived in the Congo by crossing species from chimps to humans [60]. After an infection, acquired immunodeficiency syndrome develops, resulting in increasing immune weakness and life-threatening opportunistic infections [61]. HIV infects T cells that have a vital role in immunity [62]. The nuclear material of HIV comprises two copies of single-stranded RNA transcribed into double-stranded DNA by a complex reverse transcription process [63]. Nearly 37.9 million people have caught an HIV infection worldwide in 2018, and approximately 770,000 people had died due to HIV infection [64]. Identifying biomarkers and viruses in biological matrices has broad applications in early disease diagnostics and treatment monitoring. Shafiee et al. [65] utilized nanostructured photonic crystals (PC) to capture and analyze intact HIV-1 viruses from biological samples. Researchers have also demonstrated a label-free and optical sensing technique. Upon irradiation with a broadband light source, the surface of the developed PC biosensor resonantly reflects a narrow wavelength band. The biological target adsorbs on the transducer surface, causing a shift in the resonant peak wavelength value (PWV) that can be detected with a wavelength resolution of 10 pm, allowing the identification of both biomolecular layers and a small number of viruses that are sparsely populated on the surface. The scientists were able to successfully collect and detect HIV-1 in serum and buffer samples with viral loads ranging from 10^4^ to 10^8^ copies mL^−1^. Diagnosis of HIV through a non-invasive route can be a significant way to diminish death [66].

### 2.4. Hepatitis

Hepatitis is inflammation of the liver. The disorder can be self-limiting or lead to fibrosis (scarring), cirrhosis, and liver cancer [67]. Hepatitis viruses most commonly cause hepatitis, although it can also be caused by other infections, toxic substances (such as alcohol and some medicines) [68], and autoimmune illnesses [69,70]. Hepatitis viruses are classified into five types: A, B, C, D, and E [68]. Because of the load of illness and death they inflict, as well as the potential for outbreaks and epidemic spread, these five categories are the most concerning [71]. Types B and C cause chronic disease in millions of people and are the leading cause of cirrhosis and cancer in the liver. Ingestion of contaminated food or water is the most common cause of hepatitis A and E [72]. Epidemics related to contaminated food or water can erupt explosively, such as the Shanghai (1988) epidemic that affected about 300,000 people [73]. Hepatitis B, C, and D usually occur because of parenteral contact with infected body fluids [74]. Hepatitis B transmission from the mother to baby at birth, from family member to child, and sexual contact are all common means of transmission for these viruses [75]. Jaundice (yellowing of the skin and eyes), black urine, intense exhaustion, nausea, vomiting, and abdominal pain are signs of an acute infection [76]. Hepatitis A is always a short-term, acute infection, but hepatitis B, C, and D are more likely to become chronic and persistent. Hepatitis B is spread by encountering infected blood samples, exchanging infected razors or dentist tools, and so on [77]. The hepatitis B virus has infected over 2 billion individuals worldwide (one out of three people). Every year, 30 million people become infected from the virus. Based on the evidence, nearly 292 million people are infected on a long-term basis [78]. More than 850,000 people die each year due to complications of hepatitis B infection, such as liver cirrhosis and carcinoma. Globally, an estimated 71 million people are infected with the chronic hepatitis C virus [79]. The hepatitis C virus causes hepatitis C (HCV) [80]. Hepatitis C is spread through direct contact with contaminated bodily fluids, most commonly through injectable drugs [79]. In the United States, HCV is one of the most frequent blood-borne viral infections [81]. Hepatitis D, called delta hepatitis, is a severe liver disease caused by the hepatitis D virus (HDV) [82]. HDV is contracted through direct contact with infected blood [83] and propagates hepatitis B infection [84]. An optical fluorescence-based assay was developed to detect hepatitis C viral DNA with high sensitivity and selectivity [85]. A low-cost δ-FeOOH nanosheet was used as the novel fluorescent quencher. The dye-labeled ssDNA probe was quenched by δ-FeOOH nanosheets by fluorescence resonance energy transfer due to the solid binding capability between the single-strand DNA (ssDNA) and the δ-FeOOH nanosheets (FRET). The target DNA and dye-labeled ssDNA probe would then form a double-stranded DNA complex (dsDNA) after the target DNA was added. Due to the weak bind affinity between the short fragments and δ-FeOOH nanosheets, the dye-labeled ssDNA probe in the dsDNA complex was sequentially analyzed into short fragments from the 3′-terminus by Exonuclease III, and the fluorescence signal was recovered. By exploiting these nanosheets and integrating them with exonuclease III-assisted target-recycling signal amplification, the limit of detection was improved to 10 pM [85].

### 2.5. Dengue

Mosquitoes transmit dengue virus (DENV) infection [86]; dengue virus is a single enveloped positive-stranded RNA virus with five serotypes [87]. Half of the world’s population is infected by DENV. Nearly 3 billion people are infected [88]. About 400 million people are infected yearly with DENV. Approximately 100 million people get sick from infection, and 22,000 die from dengue complications [89,90]. Avian influenza viruses can infect birds and humans. The transmission of disease occurs through contact with sick birds [91]. It can also be transmitted from an infected human to others [92]. Symptoms are very similar to the common cold, including fever, cough, sore throat, headache, muscle pain, and shortness of breath. Some antiviral drugs may be helpful if taken within two days of the appearance of symptoms [50].

Dengue E protein [93] detection based on a biofunctionalized tapered optical fiber (TOF)-based sensor with a polyamidoamine (PAMAM) dendrimer was created. The TOF dimension created an evanescent field that was sensitive to changes in the external medium, and incorporating PAMAM increased bio-recognition molecule adhesion and anti-DENV II E protein antibodies. As a result, more active sites for DENV II E protein absorption onto the tapering region were formed. With *K_d_* = 1.02 × 10^−10^ M, the sensor’s resolution and detection limit were 19.53 nm/nM and 1.0 pM, respectively. This research has presented promising potential to improve dengue diagnostics. A spin coating approach was used to create an immobilized monoclonal antibody (IgM) on gold/Fe-MPA-NCC-CTAB/EDC-NHS thin film to detect DENV E protein [94]. Upon exposure to DENV E-protein, IgM immobilized gold/Fe-MPA-NCC-CTAB/EDC-NHS thin-film generated a SPR signal in the concentration ranges from 0.0001 to 10 nM. The developed method exhibited a linear relationship between SPR angle and concentration of DENV E-protein up to 0.01 nM, with a sensitivity of 39.96° nM^−1^.

### 2.6. Biomarkers

Several biomarkers such as DNA, RNA, peptides, antibodies, glycoproteins, antigens, etc., can be utilized as an analyte of interest to detect viral infectious [95]. These biomarkers are classified as two significant categories: antigens and antibodies. Most of the stated viruses have either RNA or DNA as genetic material to encrypt proteins [5]. The viruses encompass three fundamental elements of precisely genetic material (DNA or RNA), nucleocapsid protein, and, lastly, capsid proteins. The hereditary substance is principally obscured with the encircle proteins known as nucleocapsid proteins, pursued by the wrapping of derivative envelope proteins, for instance, capsid protein. The virus admittance commences with the addition of cell-surface receptors and culminates with the release of the viral genome to the host cell cytoplasm [96]. Such access arises in two ways: endocytic and non-endocytic routes. The entire virus components, whether it is RNA, DNA, or nucleo/capsid proteins, will be instantly accessible when the virus penetrates the host cell. The B lymphocytes squirt the immunoglobulins in the body in reply to the virus antigen mechanisms. Therefore, the accessibility of the antigens (RNA or DNA, proteins) and corresponding antibodies in the neighboring cell renders it possible to distinguish by the optical biosensing methods by deploying suitable biomolecules [97,98,99]. Besides the viral diagnostics, examining the acuteness of the viral contamination is equally crucial to cure the ill person.

Due to their similar symptoms, one of the most prevalent medical challenges communities have faced is separating viral illnesses from bacterial infections, or vice versa. However, there is a 50% probability that viral infections will be misinterpreted as bacterial illnesses, necessitating antibiotic prescriptions to prevent the spread of life-threatening pathogenic germs [100]. Furthermore, an ideal biomarker must be independent of the length of febrile illness and comorbidities, as well as detectable qualities from minimally intrusive samples. The gravity of the viral disease can conceivably be evaluated by identifying other biomarkers such as interleukins, C-reactive protein, TNF- α, glutamate, interferons, D-dimer, and hematological biomarkers. Infection biomarkers can be categorized into therapeutic and pathogenetic biomarkers that play a critical role with clinical significance in diagnostics and prognostics. These biomarkers may well be measured by optical biosensing methods [101,102].

The majority of the studies are focused on identifying one or more host biomarkers that are produced as a result of the body’s immunological responses to infections. The immune system of the host reacts to infection by releasing chemicals into the circulatory system that reflect real-time pathogenic changes in the body. Because of their involvement in a variety of disease processes, the concentrations of these compounds released into the bloodstream have biological importance and hence serve as target biomarkers [103]. However, not all of these molecules are appropriate for this purpose, and they must meet particular criteria. For example, polymorphonuclear leukocytes (PMN), human neutrophil lipocalin (HNL) [104], neutrophil counts, white blood cells (WBC), and erythrocyte sedimentation rate (ESR) [103,104] are high-performing hematologic host biomarkers with statistically significant findings. PMNs, or phagocytes, have been found to be raised in the blood and are thought to play a key part in the host’s defense response during an infection episode [105]. In the event of various etiological agents that stimulate immunity, these cells inherit diverse sets of information and, as a result, operate as disease biomarkers in specific infection contexts [106]. Inflammation indicators such as C-reactive protein (CRP) and procalcitonin (PCT) indicated statistically significant differences between bacterial and nonbacterial illnesses. In humans, CRP is a Ca-dependent ligand-binding plasma protein made up of cyclic homo-pentameric non-glycosylated polypeptide subunits [107], whereas PCT is a precursor of the hormone calcitonin released by the thyroid glands C cells [108]. They are now well recognized for acute and severe viral infections, as their level is a crucial criterion for inflammation. Cytokines, which are mediators of inflammatory responses to invading pathogens and act as infection biomarkers, are another family of host protein biomarkers. Pro-inflammatory cytokines, such as IL-1, IL-2, IL-6, IL-8, and TNF, as well as anti-inflammatory cytokines, such as IL-4, IL-10, and IL-13, can be divided into two categories [102,109]. Among all, the glycoprotein (21 kDa) that makes up IL-6 is generated mostly by macrophages and lymphocytes. It is involved in lymphocyte and macrophage activation and accumulation, as well as the release of monocyte chemoattractant protein-1 (MCP-1) [110]. IL-6 levels have been found to be high in individuals with severe infection; therefore, it can be used as a disease indicator [111].

## 3. Literature Overview (State of the Art)

The influence of viruses on public health has had a significant impact on economic development in many countries. Thousands of people die every year from diseases involving a diverse variety of viruses around the world. In the last few decades, there have been growing concerns about their possible use in biological weapons [112,113,114]. Viruses can propagate quickly by natural means. Even though much is understood about viruses’ biology and pathogenicity, the advent of new agents and viruses’ ability to mutate rapidly will make them difficult to detect. Viruses remain enigmatic for a variety of reasons, making it impossible for physicians to recognize them quickly. A clinical diagnosis is difficult at this stage, and a confirmatory test is needed to identify the pathogen. Since the infecting agent may be several viruses or bacteria, a multiplexed test might be required to classify it accurately [115]. Fast, high-performance, and responsive virus identification are essential for an infected person to be adequately diagnosed and treated. The lack of adequate analysis tools and associated management interventions due to insufficient access to centralized and equipped health care facilities for diagnosis are the underlying causes of such severe diseases [116].

Significant problems for infectious disease control include the inappropriate usage of antimicrobials, the prevalence of multidrug-resistant (MDR) infections, the advent of novel infectious agents, and the speed at which disease spreads due to overpopulation and globalization [117]. A prompt diagnosis and implementation of selective antimicrobial therapy are crucial to effective infectious disease control. At the moment, a range of laboratory-based experiments, including microscopy, cultivation, immunoassays, and nucleic acid amplification, are used to diagnose clinically acute infectious diseases caused by bacterial, mycobacterial, virus, fungal, or parasite pathogens [117]. These in vitro diagnoses, though used extensively, have well-known weaknesses. In certain therapeutic cases, microscopy lacks precision, and culture has a considerable wait. Although highly responsive, immunoassays such as ELISA are labor-intensive and difficult to introduce. Nucleic acid amplification experiments, such as PCR, give molecular precision but have complicated sampling and false positives potential. The standard procedure flow to the common diagnosis of infectious diseases involves selecting and transferring biological samples from the treatment point to a central laboratory for expert sample analysis. Upon access to the findings (typically in days), the lab informs the physicians who contact the patients and modifies the procedure if required. This intrinsic inefficiency makes the provision of evidentiary treatment more complex at the right time and harms the use of antimicrobials [117].

A biosensor is an analytical tool that transforms molecular recognition of a target analyte to a measurable response through a transducer [118]. Biosensors are making a big difference by restructuring their sensing module for biomolecular detection, nanosized products, such as protein biomarkers and viruses, to convert the standard current analytical protocols into diagnostic strategies. Because of their high sensitivity to many well-known optical phenomena, such as surface plasmon, scattering, and interferometry, optical transducers are commonly used [119]. Optical biosensors, which can track and recognize chemical and biological organisms by calculating complete reflection as a recognizing signal (changes in absorbance, fluorescence, luminescence, polarization, and refractive index) with photodetectors, are important alternatives to conventional analytical techniques [120,121]. In comparison to other physicochemical transducers, SRP transducers have exciting features that allow for the monitoring of analytes in real-time without labeling. The immobilized bio transducer interacts with the analyte, causing a local rise in the metal surface refractive index to support an SPR signal change [122,123].

Current studies focus on developing methodologies for single-analyte, multi-analyte with smartphones and biochip microfluidic device integration [102]. Several optical biosensor systems for bacterial and viral biomarker detection have been developed, in which an immunosensor have been inserted into microfluidic platforms such as leukocytes from biological samples [124]. A biosensor-chip designed to caption the leucocytes of a biological specimen by antigen-antibody interactions is made from polydimethylsiloxane (PDMS), which is on a glass slide with thick microfluidic channels. The signal from the biochip is collected using a smartphone through fluorescent imaging and quantified using image processing software, which is appropriate for cell-limited resource biosensing infections [124]. A plethora of literature has already been published on monolithic integration for the detection of microbes [125,126,127].

Prilutsky et al. [116] presented an optical chemiluminescence-based assay to describe the functional activity of polymorphonuclear leukocytes (PMNs) or phagocytes. The reaction byproducts were examined using chemiluminescence (CL) measurements to quantify and localize respiratory burst generation. PMNs from the individuals with acute infections of the whole-blood system were subjected to this technique, which required luminol amplification for CL measurements. CL is thought to be a powerful means of exploring phagocytes’ oxidative potential, and it may be evaluated as a luminol-dependent CL. This technique was used to identify viral or bacterial illnesses in origin using blood samples from 69 individuals with a fever (>38 °C).

Surface plasmon resonance (SPR) is the excitation of an electromagnetic wave that propagates by a certain angle of incident light beam through the interface of two media with dielectric constants of other signs, such as metal and a sample buffer [128,129]. The signal is dependent on absolute internal absorption, which causes the reflected light to have a lower intensity. Chen et al. were the first group to use a SPR biosensor for SARS-CoV morphological study [128]. SPR was capable of confirming that the N-terminal removed proteinase dimer adopts a state unlike that of the full-length proteinase dimer [128].

Any change at the interface, such as changes in refractive index or the development of a nanoscale film thickness owing to surface molecule interactions, affects the angle at which the resonance occurs.

The resonance angle is vulnerable to some alteration at the interface, such as variations in refractive index or the forming of a nanoscale film thickness due to surface molecular interactions.

As a result, these shifts can be calculated by observing the minimum change of light intensity over time. Using T4 and BP14 bacteriophages as trapping components, a bioanalyzer based on SPR was used to detect *Escherichia coli* O157:H7 and methicillin-resistant *Staphylococcus aureus* (MRSA) [130]. A SPR bioanalyzer could detect as few as 10^3^ cfu/mL in less than 20 min without labeling or enrichment. Backscattering interferometry (BI) is another optical detection way applied for biosensing [131]. BI devices consist of a coherent single-wavelength light source, which focuses on a microfluidic canal and detectors to analyze reflected strength (commonly the low-power He-Ne or red diode laser). A strongly modulated interference pattern is generated due to sub-wavelength structures in the channel following a consistent laser illumination of the fluids-filled channel. The analysis of variations in the profile of fringe patterns by the detector positioned in the immediate backscatter direction aids in calculating refractive index changes and quantifying molecular binding events [117].

Kussrow et al. have revealed the importance of applying BI for quick detection of purified total human IgG from seropositive syphilis patients using a purified recombinant treponemal antigen r17, representing the concept of using this method for serological detection in medical models [132].

Most label-free optical biosensors necessitate specific light coupling to the sensing field, which is a significant disadvantage for point-of-care applications. As a result, when this technique is used in an integration system, optical detection can be significantly enhanced [117]. Integrated optics permit multiple passive and active optical components to be placed on the same substrate, allowing for the modular construction of small, lightweight sensing devices and the manufacturing of multiple sensors on a single chip.

A dual-functional plasmonic biosensor integrating the plasmonic photothermal (PPT) effect and localized surface plasmon resonance (LSPR) sensing transduction were applied by Qui et al. as an alternate and promising approach for clinical COVID-19 diagnosis [133]. The sensor was modified with 2D gold nano-islands (AuNIs) and functionalized with complementary DNA (cDNA) receptors by forming Au–S bonds between the AuNIs and the thiolic groups of cDNA. No matter which approach is used in optical biosensors, biomarkers such as antigens or microRNAs can be accurately and rapidly detected. It is essential to reduce detection limitations (LODs) in order to identify a few analytes operating in cancer diagnosis [120]. Kaja et al. used an optical biosensor constructed regarding ovarian cancer biomarkers detection (fibronectin, apolipoprotein A1, and TIMP3). This progress is particularly significant in early diagnosis as <20 percent of ovarian cancer can be diagnosed in the early stages [134]. A photonic biosensor based on the silicon “lab-on-a-chip” for early-stage cancer has shown a sensitivity of at least 10 to 100 times greater than that of current commercial biosensors [135]. Shafiee et al. have demonstrated a mechanism free of etiquette optical sensing by nanostructured photonic crystals (PC). In resonant light with a broadband light source, the nanoplastic surface of the PC biosensor is a short wavelength band [65]. Surface-adsorbed bio targets cause a change in the resonant peak wavelength value (PWV), which can be detected with a resolution of <10 p.m., allowing both biomolecular layers and small numbers of viruses to detect that diminishing surface content of the transducer.

## 4. Safety and Security

Researchers must be mindful that infecting animals can add a new dimension to the risks, whether intentionally or unintentionally. All necessary permissible regulations and measures, including infectious agents, must be followed both before and after the experiment during an investigation. The safety and security measures for infectious agent experimentation must be approved by government policies or an in-house formed authority.

Take the necessary precautions and safety measures when working with biohazards, such as limiting access to the area to listed personnel only, decontaminating all surfaces and waste after each day of experimentation, not pipetting or spitting during an investigation, wearing personal protective equipment (including an eye mask and face shield), being familiar with the written instructions and documentation of the working area or experimentation, and adhering to the waste disposal procedures [136].

## 5. Objective of the Present Review

**Figure 1:**
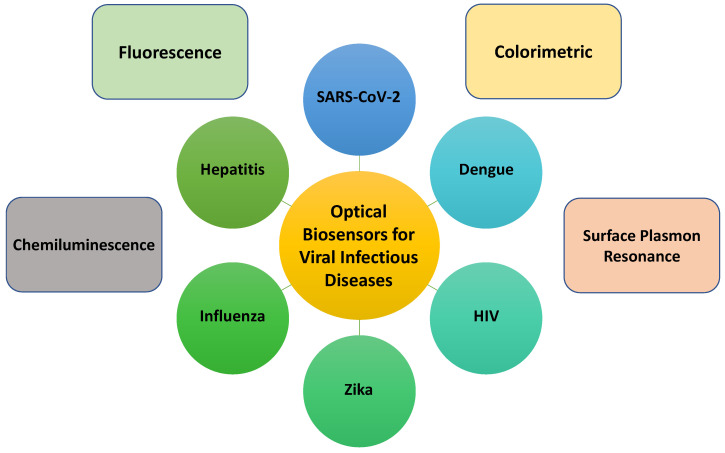
Schematic representation of the optical biosensors in viral infectious disease diagnostics.

The current review discusses the latest advancements in the optical biosensors of human viral infectious diseases. This review focuses on the optical detection of COVID-19, MERS, SARS, Influenza, Hepatitis, HIV, HPV, Zika, Herpes simplex virus, Chikungunya, Dengue, and Rotavirus. All-important optical works are divided into sub-sections to better comprehend the sensor fabrication’s primary idea/principle/working mechanism. The first section summarizes recent publications on optical biosensors for the detection of numerous life-threatening viruses. The following section exclusively discusses the various optical sensor systems, including calorimetrically, fluorescence, chemiluminescence, surface plasmon resonance, and photonic transduction methods. The key obstacles in transferring laboratory research into real-world device applications are described in detail. Optical sensors for viral detection are discussed in terms of prospects and commercialization. The background and overall progress presented in this paper can aid researchers in devising new novel ways to construct point-of-care optical diagnostic sensing systems for various pathogens, not just life-threatening viruses. Figure 1 conveys the overall summary of optical sensors towards the detection of the abovementioned selected infectious diseases.

## 6. Optical Diagnostics

Optical methods for virus detection have recently gained popularity due to their advantages of being fast, label-free, reusable, inexpensive, and portable for use at the point of care (POC) [34]. Noninvasive optical methods often preserve virus viability, making them useful for researching virus transmissibility, virulence, evolvability, and immunology [137]. Epitope variation, for example, may result in escape mutants being selected under immune strain, rendering either a possible detection tool or inactivation of vaccine [138,139]. The current state of art techniques in colorimetric, surface plasmon-based, fluorescence, and chemiluminescence have enabled faster, more sensitive detection with a low limit of detection [140,141,142,143,144].

### 6.1. Colorimetric Sensors

**Figure 2:**
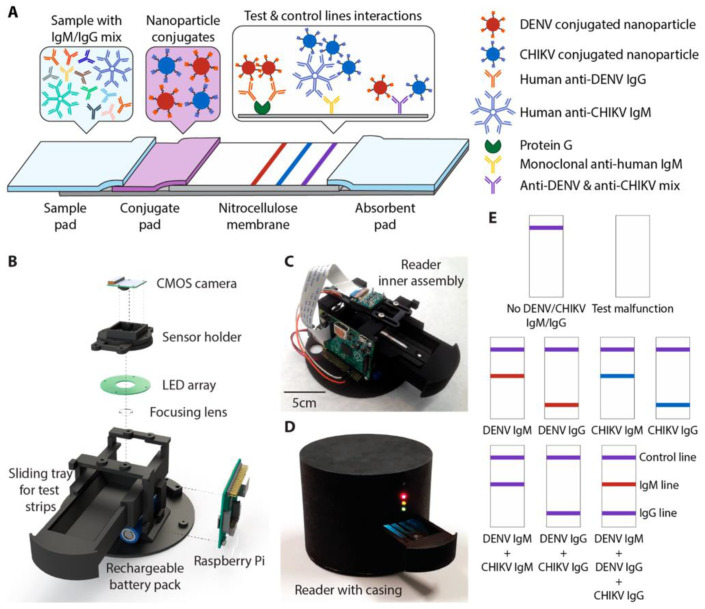
A 4-plex color encoded lateral flow test strip and an optical scanner make up this quick diagnostic platform. (**A**) Multiplex lateral flow test strip architecture. DENV and CHIKV antibodies can attach to red and blue nanoparticle conjugates, respectively. The first test line (red) captures nanoparticle conjugates labeled IgG, while the second test line (blue) captures labeled IgM, resulting in color development. When both DENV and CHIKV Ab of the same isotype are present in a sample, the test line generates an intermediate hue that is a mix of red and blue. The control line catches both unbound nanoparticle conjugates and unbound nanoparticle conjugates (purple). (**B**) An optical reader with an exploded view is designed to reduce color detection variations. (**C**) The optical reader’s internal assembly. (**D**) is fully built and protected from the elements. To avoid ambient light, the open sliding tray is closed during imaging. The lights on the indicator show the progress of imaging. (**E**) Illustrations of test strip appearance for various diagnostic circumstances. DENV and CHIKV are both present in purple cases. Ab has the same isotype (adapted with permission from Wang et al. 2019 131, Copyright © 2019, American Chemical Society) (adapted with permission from Wang et al. 2019 [141], Copyright © 2019, American Chemical Society).

Sensors’ technology utility in detecting a wide range of analytes quickly and efficiently has grown dramatically in recent years. The colorimetric sensors with a simple platform, quick response, fair sensitivity, and selectivity are among the most powerful and impressive devices for detecting microbes, biomolecules, and emerging contaminants [145,146]. In colorimetric biosensors, a complex ligand-target interaction results in a shift in color that can be seen with both the naked eye or be used as simple portable optical detectors for quantitative measurement [147,148]. It may be a solution or solid support. Rahman et al. [149] reported the development of a solution-phase bioassay protocol for the detection of dengue virus (DENV). This study demonstrated a label-free optical technique based on DNA/PNA hybridization and unmodified gold nanoparticles (AuNPs). In this approach, the unmodified AuNPs undergo an aggregation with neutral peptide nucleic acid (PNA) sequences, as they adsorb on the surface of AuNPs in the absence of targeted dengue virus. It showed a change in color from red to purple and resulted in a shift in wavelength from 520 to 650 nm, which is due to the aggregation process of free PNA molecules and AuNPs without the addition of NaCl (5 M) [150]. With the addition of complementary dengue DNA, the G-quadruplex complex forms due to hybridization of the targeted DNA and PNA probes, and the AuNPs remained suspended and did not form aggregates. The color change was observed with a naked eye and with the UV–vis spectrum as well. A detection limit of 0.12 μM for the PNA: DNA ratio (1:0.01) was calculated. Recently, a rapid diagnostic platform integrated with a low-cost reader and a multicolor 4-plex immunoassay was demonstrated to detect and distinguish between DENV IgM and IgG. With a detection time of 30 min, the developed platform was useful in quantifying DENV and chikungunya virus (CHIKV) and IgM and IgG antibodies in human clinical samples (Figure 2) [141]. This multiplex assay possesses many advantages, such as a low sample volume and the ability to detect four analytes simultaneously over traditional rapid diagnostic tests used in resource-constrained environments. The Hook effect was neutralized in this study using a simple dilution approach [151].

**Figure 3:**
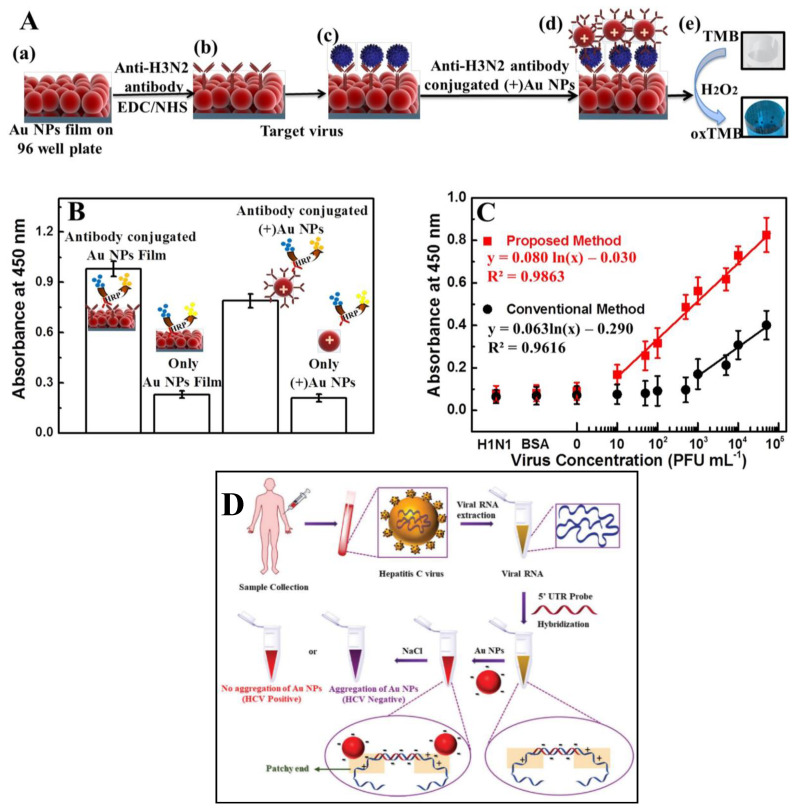
Design and detection of Influenza virus A (New Caledonia/20/1999) (H1N1) was discovered on film. (**A**) Virus detection schematic: (**a**) AuNP films on 96-well polystyrene plates; (**b**) anti-HA antibody immobilization on AuNPs using EDC/NHS chemistry. (**c**) The target virus was introduced after washing the NPs (n = 3) and incubated at room temperature for 30 min. (**d**) The virus was bound by the anti-NA antibody-conjugated (+)AuNPs via an antibody-antigen reaction, and the unbound (+)AuNPs washed away. (**e**) TMB-H2O2 was added, and the oxidation of the peroxidase substrate TMB resulted in fast color shifts (oxTMB). (**B**) Anti-NA antibody binding to (+)AuNPs via electrostatic interactions as determined by ELISA. (**C**) The absorbance calibration curve for the influenza virus A concentration (New Caledonia/20/1999) (H1N1). H3N2-donated influenza virus A/Yokohama/110/2009 (H3N2) was used to test the system’s specificity (n = 3) (adapted with permission from Ahmed et al., Scientific Reports, 2017 [156]). (**D**) Representation of the AuNP-based assay strategy for HCV viral RNA sensing in clinical samples (adapted with permission from Mohammad et al., Analyst, 2021 [160]).

The binding affinity of hemagglutinin (HA) protein present on the surface of viral species with Sialic acid has been reported as one of the critical determinants for various viral species such as influenzas [152,153,154]. Conceptualizing the binding affinity of sialic acid to the viral surface, Lee et al. [155] proposed a colorimetric-sensing strategy utilizing sialic acid modified AuNPs to determine the influenza virus. A green method was used to synthesize and stabilize AuNPs with a size of 20.10 ± 1.80 nm. An increase in optical density and dilution of chemically inactivated influenza B virus exhibited an excellent linear relationship. The viral dilution of 0.156 vol% was readily detectable. However, the linearity can be further improved by using a higher concentration of sialic acid-stabilized AuNPs. In a recent study, a simple strategy for the in situ production of AuNPs films by mixing sodium formate and chloroauric acid (HAuCl_4_), which can be implement on various surfaces, such as a 96-well plate, PDMS, etc. [156] was reported. The peroxidase-like catalytic activity of Au NP films and colloidal (+)Au NPs against TMB-H2O2 mixtures was used to establish a responsive and quantitative method for colorimetric detection of influenza virus A (H1N1) (Figure 3A). An ELISA was used to confirm the conjugation of the anti-NA antibody with (+)Au NPs (Figure 3B). A linear range from 10 pg mL^−1^ to 10 µg mL^−1^ (Figure 3C) with LOD of 50.50 pg mL^−1^ was established for New Caledonia/H1N1/1999 influenza virus, whereas, for clinically isolated influenza A virus (H3N2), a LOD of 24.3 PFU mL^−1^ with a viral range from 10–50,000 PFU mL^−1^ was demonstrated in a concentration-dependent manner. On a glass substrate, the developed platform exhibited a LOD of 4.5 pg mL with linearity up to 10.0 pg mL^−1^ for avian influenza virus A (H5N1/ Vietnam 1203/04). A smartphone-integrated point-of-care platform utilizing AuNPs and ZnO nanorods reported the detection of avian virus [157]. This platform allows the detection of 2.7 × 10^4^ EID_50_/mL with the naked eye, whereas a smartphone imaging system improves the limit down to 8.0 × 10^3^ EID_50_/mL, with a total time of 1.5 h required to complete the detection process. This developed platform with an integrated microfluidic system and smartphone-enabled plasmonic-based colorimetric detection possesses the inherent advantages of a portable, robust, fast, and user-friendly platform with higher sensitivity. Furthermore, this could help in the critical needs in low-resource areas for the monitoring of viral infection.

The detection of DNA molecules plays a vital role in the early diagnosis of genetic diseases. However, the conventional approaches rely on expensive equipment, which does not meet the demands of developing countries. A copper nanocluster-based colorimetric sensor for detection of the hepatitis B virus was published by Mao et al. [158]. The method showed excellent sensitivity and could detect viral load down to 12 × 10^9^ molecules. The UV–vis absorbance at 420 nm (the maximum absorption of oxidized ABTS) and the logarithm (log) of target DNA concentration showed a good correlation, with increasing DNA concentration and observed UV absorption. The linearity of measurement was established between UV–vis absorbance at 420 nm (the maximal absorption of oxidized ABTS) and logarithm concentration of target DNA from 0 to 12.0 × 10^13^ molecules with a correlation coefficient of R^2^ = 0.99 when concentration was increased from 12.0 × 10^9^ to 12.0 × 10^13^ target molecules. It was evident from the results that the addition of at least 12 × 10^9^ target DNA molecules was required for catalytic activity, which confirms the detection limit of 12 × 10^9^ molecules for analysis. For an early diagnostic of HBV infection, a colorimetric immunosensor was constructed utilizing conjugating magnetic separation and enzymatic catalysis [159]. This platform showed a relationship with an increase in HBsAg concentration in the 0.10–20 ng mL^−1^ ranges and OD450 nm with a detection limit of 0.012 ng mL^−1^. Furthermore, the performance of the immunosensor was also quantified in real human serum samples.

In another work, Mohammad et al. proposed a susceptible and selective colorimetric biosensing platform for detecting hepatitis C virus [160]. With these considerations in mind, a straightforward strategy was devised to design a non-thiolated antisense oligonucleotide complementary to the 5′ UTR of the HCV viral genome. This 5′ UTR probe was explicitly designed to target HCV RNA in clinical samples. The interaction of citrate-capped Au NPs with a combination of the 5′ UTR probe and HCV viral RNA was studied in terms of Au NP stability (Figure 3D). The use of non-thiolated complementary oligonucleotides sequence avoids the chemical reaction for the conjugation of gold-thiol. This AuNP-based assay showed a vast range of 7.50 × 10^2^ to 2.00 × 10^6^ IU mL^−1^ viral load for HCV RNA detection in clinical samples with a limit of detection of 100 IU mL^−1^ (0.4 IU µL^−1^) and turned-out time of ~30 min. Because of its high sensitivity and selectivity, the assay can be used for early and acute infection detection and disease control. Other viral diseases, such as SARS, Middle East respiratory syndrome (MERS), HIV, COVID-19, and others, can benefit from the strategy mentioned here.

Very recently, utilizing a collection of in-house built initiators targeting the region encoded the N protein, a loop-mediated isothermal amplification (LAMP) method was demonstrated to detect and amplify SARS-CoV-2 genetic sequences by González et al. [161]. Visual inspection was implemented to distinguish between positive and negative samples in this LAMP-based technique. They used color decomposition and analysis in the color CIELab space to quantify color differences between positive (yellow) and negative (red) samples. Furthermore, the sensitivity of this LAMP colorimetric system was compared to PCR protocols. This basic strategy could be sufficient for quickly deploying diagnostic efforts in the event of a COVID-19 pandemic. This strategy could detect and amplify SARS-CoV-2 nucleic acids in the range of 625 to 2 × 10^5^ DNA copies with 92.85% sensitivity and 81.25% specificity when 44 RNA extracted from patients were analyzed in a hospital.

Meanwhile, a colorimetric biosensor reported by Ventura et al. based on SARS-CoV-2-induced gold nanoparticle (AuNP) interaction proves to be an excellent method for detecting viral particles in nasal and throat swabs [162]. The extinction spectrum is red-shifted quickly when a colloidal solution of several viral-target gold nanoparticles (AuNPs) functionalized with antibodies targeting three surface proteins of SARS-CoV-2 (spike, envelope, and membrane) combined with a solution containing the viral particle. When the optical density of the mixed solution was calculated at 560 nm and compared to the threshold cycle (Ct) of a real-time PCR (the gold standard for detecting the presence of viruses), it was observed that the colorimetric method detected very low viral load, with a similar detection limit to the former one. Another exciting aspect of the biosensor reported here is that it is sensitive to the virion rather than the content of the virion (RNA). The significance of this is twofold: first, after the optical response is calibrated, the biosensor becomes a potent instrument for quantifying viral load, a non-trivial issue in virology diagnostic procedures. Second, because the biosensor is exclusively sensitive to virions, it detects the presence of active viral particles; hence, our approach is suitable for determining the degree of infectivity of a sample.

From this study, the published results opened a new perspective in the sense of current and potential future pandemics and microbiology, as the biosensor demonstrates itself to be a powerful but easy tool for measuring viral particle concentration. Table 1 summarizes the colorimetric-based sensing platform reported for various kinds of viral infections.

### 6.2. Fluorescence/Chemiluminescence-Based Approaches

A fluorophore substance is excited by a particular wavelength in a standard fluorescence measurement and then emits light at a different wavelength. The sensitive identification of the desired analytes present at a trace level in the sample necessitates the use of reporter molecules labeled with fluorescent dyes [167]. High detection sensitivity (single-molecule detection), quick response times, a localized fluorescence signal, multiplexed assays using multicolor dyes, and an uncomplicated labeling method that produces suitable functional groups on the target are all advantages of fluorescence detection [168].

**Figure 4:**
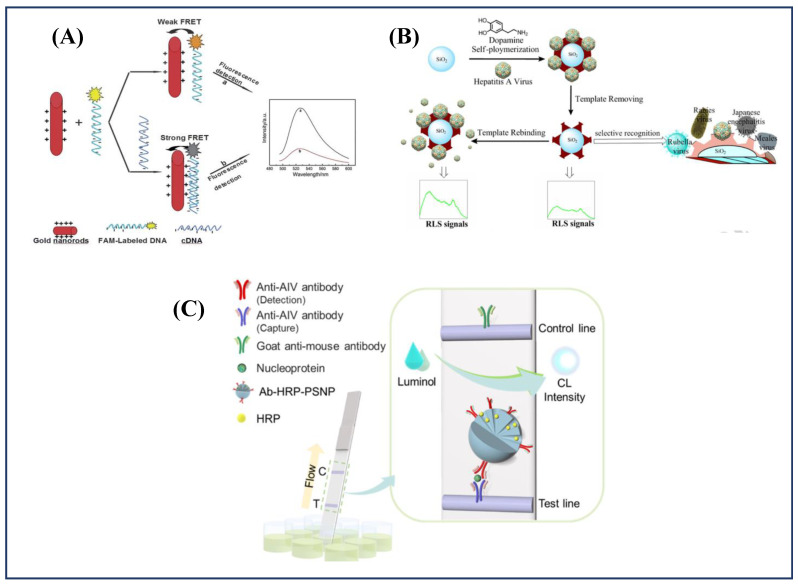
(**A**) Schematic representation of the strategy for DNA hybridization detection (adapted with permission from Lu et al., Analyst, 2013 [169]); (**B**) Principle of preparation of the virus-MIPs and detection of virus (Hepatitis A) (adapted with permission from Yang et al., Biosensors, and Bioelectronics, 2017 [171]); (**C**) Schematic illustration of porous silica nanoparticle-based chemiluminescent lateral flow immunoassay (CL-LFA) platform for the detection of avian influenza virus (AIV) nucleoproteins (adapted with permission from Jung et al., Analytical Chemistry, 2021 [178]).

To quantify hepatitis B viral DNA sequences, a FRET-based biosensor was designed using gold nanorods (AuNRs) and the fluorescein (FAM) molecule [169]. The AuNRs were generated using a seed-mediated surfactant method and then modified with cetyltrimethylammonium bromide (CTAB) to produce positively charged AuNRs. Figure 4a depicts the virus identification technique applied. When single-stranded FAM-labeled DNA was added to the AuNRs suspension, the DNA sequences adsorbed onto the positively charged AuNRs’ surface. Fluorescence quenching occurs due to the development of the FAM-ssDNA–CTAB–AuNRs ternary complex and the FRET process from FAM to AuNRs. When complementary target DNA was added to the FAM-ssDNA–CTAB–AuNRs complex solution, the fluorescence intensity was reduced even more due to improved quenching effectiveness. Over a working range of 0.045–6.0 nmol L^−1^, the biosensor has a detection limit of 0.015 nmol L^−1^ (n = 3). Shen et al. [170] reported an ultrasensitive method to detect hepatitis B virus surface antigen (HBsAg) in human serum. It employed a sandwich immunochromatographic assay (ICA) based on the signal amplification capability of highly luminescent quantum dot-beads (QBs) [170]. The sensor demonstrated a detection limit as low as 75.0 pg mL^−1^ HBsAg in the working range of 75.0 pg mL^−1^–75.0 ng mL^−1^. Yang et al. [171] proposed a new resonance light-scattering (RLS) sensor based on mussel-inspired hepatitis molecularly imprinted polymer to precisely detect trace quantities of Hepatitis A Virus (HAV). In this work, a polydopamine (PDA)-coated totivirus-imprinted polymer was introduced on the surface of SiO_2_ nanoparticles (virus-imprinted SiO_2_@PDA NPs) as a recognition factor using an effective one-step syringe method (Figure 4b) with a low detection limit of 8.6 pmol L^−1^. The enhanced RLS intensity (IRLS) was proportional to the concentration of HAV, in the range 0.04–6.0 nmol L^−1^.

For the sensitive detection of recombinant hemagglutinin (rHA) protein of the H5N1 influenza virus in human serum, a fluorescent aptasensor based on core-shell nanoparticles metal-enhanced fluorescence for H5N1 influenza virus detection (MEF) was designed [172]. Guanine-rich anti-rHA aptamers produced by SELEX were immobilized on the surface of Ag@SiO_2_ nanoparticles, which served as a metal-enhanced fluorescence (MEF) sensing tool. The fluorescent tag thiazole orange (TO) was used to report the G-quadruplex secondary structural induced by the aptamer-rHA binding case. The rHA protein of the H5N1 influenza virus was observed in aqueous buffer and human serum, with detection limits of 2.0 and 3.5 ng mL^−1^, respectively. Nonetheless, the issue of cost-consuming production and antigenic drift interference could be solved by using this aptamer-based biosensor, which is inexpensive and easy to use. More significantly, the entire detection process in a PE tube can be completed in 30 min, making it a self-contained diagnostic kit for H5N1 influenza virus point-of-care (POC) diagnostic. Interestingly, a turn-on sensor constructed integrating tetraphenylethylene derivatives (a fluorescent probe) was proposed by Kato et al. [173]. The system used to manufacture these probes has the advantage of allowing ligands added to probe compounds through “click chemistry,” a Cu(I)-catalyzed azide-alkyne cycloaddition method that can speed up the manufacturing process. A concentration of 10^5^ pfu/100 µL influenza virus was easily detected. Fukuyama et al. [174] reported the development of a multi-spectral fluorescent reporter influenza viruses (Color-flu) as powerful tools for in vivo studies. Color-flu viruses are powerful tools for analyzing fatal virus (H5N1 and H7N9) infections at the cellular level in vivo to understand influenza pathogenes.

Yang et al. published a three-dimensional Cu-based metal-organic framework-1 (MOF) that is water stable and supported by a tritopic quaternized carboxylate and 4,4′-dipyridyl sulfides as an ancillary ligand [175]. With detection limits of 196 and 73 pM, respectively, this device can be used as effective fluorescent sensors for human immunodeficiency virus one double-stranded DNA and Sudan virus RNA sequences. Very recently, a triple-mode (colorimetric/SERS/fluorescence) biosensor based on AuNPs was recently proposed for the rapid and selective detection of RNA in SARS-CoV-2 in 40 min [176]. Colorimetric, surface-enhanced Raman scattering (SERS), and fluorescence signals of sensors were observed simultaneously based on their specific aggregation property and affinity energy to distinguish AuNPs with an average size of 17 nm. The sensor achieves a femtomole level detection limit of 160 FM in absorbance mode, 259 FM in fluorescence mode, and 395 FM in SERS mode in all triple modes. The triple-mode sensor’s signals can detect the single-base mismatch, reduce SARS-CoV-2 false negative readings, and offer a new method for detecting COVID-19 and other fast, responsive, and selective diseases.

To detect anti-DENV IgM antibodies, an indirect chemiluminescence enzyme immunoassay (CLEIA) based on synthetic peptides selected from the envelope protein of DENV using computational calculations was reported by Zhu et al. [177]. For detection of avian influenza viruses (AIV) and other infectious and fatal viral avian-origin diseases, a signal-amplifiable nanoprobe-based chemiluminescent lateral flow immunoassay (CL-LFA) was developed by Zung et al. [178]. In the approach, antibodies (binding receptors) and enzymes (signal transducers) can be immobilized on sensitive paper-based sensor platforms using size-selective signal amplifiable nanoprobes (Figure 4C). The CL-detection LFA’s limit for the nucleoprotein of the H3N2 virus using the signal-amplifiable nanoprobe was reported to be 5.0 pM. The CL-LFA exhibited higher sensitivity and specificity for detecting AIV in clinical specimens of swab samples taken from contaminated chicken hosts.

HTLV-II (Human T-lymphotropic Virus Type II) is a type-C retrovirus linked to several human diseases [179]. For the first time, the fabrication of a bio-bar code for dendritically amplified HTLV-II DNA sensing was reported by Wang et al. [180]. This method could detect HTLV-II DNA with a detection limit as low as 0.50 µM and nine magnitudes higher dynamic range from 1.0 µM to 1.0 nM. The three main reason for the above outcomes were: terminal deoxynucleotidyl transferase’s (TdT’s) efficient polymerization reaction, bio-bar-code amplification (BCA’s) power, and G-rich DNAzyme-driven chemiluminescence’s high sensitivity. For practical application, this strategy has the potential to distinguish between single-base mismatches and assess HTLV-II DNA in both human serum and human T-lymphocytic leukemia cells. A novel Pseudorabies virus (PRV) detection method based on MBs and chemiluminescence, with an intrinsic ability to detect as low as 100 amol PRV, was proposed by Yang et al. [181]. When compared to conventional PRV detection technologies, it offers a clear advantage. This method can be used in the swine industry for early detection of porcine pseudorabies due to its low cost, ultrasensitivity, and speed.

For quantitative determination of human serum HBsAg, a rapid capillary chemiluminescence immunoassay (CCIA) using a portable analyzer was established [182]. For application in clinical analysis, the sensitivity and specificity of the CCIA were validated in positive HBsAg (HBsAg+) and negative HBsAg (HBsAg) human sera. Under the optimized experimental condition, the CCIA quantitatively detected human serum HBsAg levels at 0.4–15.0 ng mL^−1^, resulting in dose-dependent increases in chemiluminescence (CL) signals with a sensitivity of 0.3 ng mL^−1^ HBsAg. Similarly, for HBsAg, a quick, sensitive chemiluminescence immunosensor with a broad linear range was developed [183]. Herein, the integration of AuNPs as carriers for luminol and secondary antibody resulted in signal amplification and a high specificity for HBsAg. Furthermore, Au^3+^ was used as a non-enzymatic catalyst to increase sensitivity. This platform was able to detect as low as 14 pg mL^−1^ HBsAg in human serum.

The first line of defense against infectious disease is to locate infected patients as soon as possible. Addressing the problem of unavailable solutions for an early and accurate quantification of SARS-CoV-2, various strategic approaches based on chemiluminescence platforms have been recently reported [184,185,186,187]. Compared to false-negative results due to viral evolution in reverse transcriptase-PCR (Rt-PCR) results, the above platforms could measure SARS-CoV-2 as quickly and accurately as possible. Moreover, the applicability of these platforms to detect SARS-CoV-2 in the clinical sample has also been reported. For example, a dual lateral flow optical/chemiluminescence immunosensor for rapidly detecting salivary and serum IgA was identified in SARS-CoV-2 infected patients [188]. Table 2 summarizes the fluorescence and chemiluminescence platforms developed for the detection of viral antigens or antibodies.

### 6.3. Surface Plasmon Based Approaches

Longitudinal electron concentration waves produced on the metal-dielectric interface by p-polarized light incidence are known as surface plasmons (SP). Matching a momentum between an incident photon and the SP causes the phenomenon of resonance. SPR biosensors that detect and measure molecular interactions are based on surface plasmon resonance (SPR), which is sensitive to surface states [131]. However, in real-time, there are no labels. The existing literature witnessed the development of various SPR-based biosensors for viral diagnostics [140].

An SPR-based sensing platform was constructed utilizing the random nanoislands to excite and localize SPs and control associated evanescent nearfields for enhanced detection sensitivity for viral particles detection [191]. The theoretical and functional data presented in this paper, on the other hand, do not reflect the best possible solution or any change in LOD or sensitivity. Furthermore, because of the limitations in computational models of nanoislands, theoretical analyses tend to be negative. Using immobilized recombinant Ebola nucleoprotein (EBOV-rNP) on a 4-mercaptobenzoic acid (4-MBA/Au)-modified SPR gold chip (EBOV-rNP/4-MBA/Au), an SPR technique was developed for screening different monoclonal antibodies (mAb1, mAb2, and mAb3) of EBOV in real-time [192]. The 4-MBA/Au SPR chip formation was characterized by SPR, electrochemical impedance spectroscopy (EIS), and attenuated total reflectance Fourier–transform infrared spectroscopy (FTIR-ATR). Among the three antibodies used, the best antibody was chosen based on the affinity constant and used to detect EBOV-rNP in a phosphate-buffered saline (PBS) medium. The interaction of Ebola mAb1, mAb2, and mAb3 with immobilized EBOV-rNP was found to have KD values of 809 nM, 350 pM, and 52 pM, respectively, indicating mAb3’s high affinity. With mAb3, the SPR limit of detection for EBOV-rNP was 0.5 pg mL^−1^.

**Figure 5:**
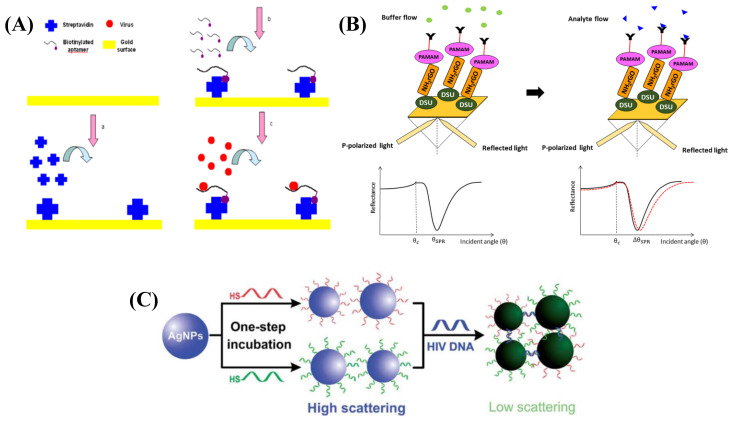
(**A**) Principle of SPR biosensor for detection of AIV H5N1: (**a**) Streptavidin immobilization; (**b**) Biotinylated apTable; (**c**) Virus detection. 2012. @MDPI [193]); (**B**) Schematic illustration of the SPR signal before and after the analyte flow (Adapted from Omar et al., Scientific Reports, 2020 [197]); (**C**) Schematic demonstration for the preparation of DNA–AgNP conjugates through one-step conjugation chemistry and a sandwich strategy for HIV DNA detection. Red (DNA1) and green (DNA2) strands represent 2 probe DNAs that can hybridize with HIV DNA (Adapted with permission from Liu et al., Analyst, 2012 [200]).

Using a selected aptamer as the recognition factor, Bai et al. proposed the development of an SPR-based biosensor for the detection of avian influenza virus (AIV) H5N1. The sensing system was created by streptavidin–biotin-binding to immobilize a biotinylated aptamer over a streptavidin-coated gold surface [193] (Figure 5A). The RI value was linearly connected (R^2^ = 0.99) to the concentration of AIV in the range of 0.128 to 1.28 HAU (HA unit) after optimizing the streptavidin and aptamer parameters. This aptasensor found AIV H5N1 in poultry swab samples with concentrations ranging from 0.128 to 12.8 HAU in 1.5 h. An inhibition assay using HA protein deposited over the sensor chip to identify whole viruses was used to quantify AIV H1N1 and H3N2 [194]. This SPR-based optical fiber sensor’s detection surface was prepared using a plasma modification method for chemically binding a self-assembled monolayer of isopropanol to the gold surface of the optical fiber. Preliminary research revealed susceptible virus detection in the range of 0.5–10 µg mL^−1^ and increased precision with a response time of 10 min. Quantification of HA via single-radial immunodiffusion (SRID) quantification is the most popular approach for ensuring product potency in seasonal influenza vaccines. Based on the above phenomenon, a fiber optic SPR-based sensing strategy to detect AIV subtype H6N1 has been reported [194]. The method uses an inhibition assay format with HA proteins for H1N1, H3N2, and B immobilized on a sensor chip to quantify the virus. In comparison to SRID, preliminary results showed that the assay had higher sensitivity (detection range 0.50–10 µg mL^−1^), precision, analysis, and hands-on time. The detection limit of <0.50 µg mL^−1^ was quantified. Lepage et al. [195] demonstrated an innovative quantum-well SPR-based configuration for real-time AIV A diagnostics. As compared to a traditional prism-based SPR design, it revealed a time resolution of 2.2 s for data acquisition, yielding a resolution of 1.5 × 10^−6^–2.7 × 10^−5^ RIU. H5Nx whole virus detection using a highly sensitive and specific sandwich-type SPR-based biosensor reported by Nguyen et al. [196]. Herein, in a Multi-GO-SELEX process, aptamers sequences were successfully screened and characterized for whole avian influenza (AI) viruses, H5Nx, for the first time. The aptasensor was efficient in the range of 8 × 10(4) to 1 × 10(4) EID50/mL with a detection limit of 200 EID50/mL for H5N1 virus.

A study on an SPR-based approach for dengue diagnosis used DENV E-protein as a target and IgM antibodies as a ligand [197]. Figure 5B shows a schematic representation of the proposed Au/DSU/NH2rGO-PAMAM/IgM sensor with DENV-2 E-proteins added. The sensor operating range was determined to be 0.08 pM to 0.5 pM, with a detection limit of 0.08 pM within 8 min with sensitivity and binding affinity values of 0.2576° pM^−1^ and 6.6844 TM^−1^, respectively. The immobilization efficiencies of variously changed surfaces were compared using model proteins with different surface charges, such as streptavidin, due to the high protein-immobilization efficiency of the plasma-treated parylene-N film [198]. Herein, an SPR biosensor based on the plasma-treated parylene-N film was developed to detect human hepatitis virus surface antigen (HBsAg), and the plasma-treated parylene-N film was estimated for improved sensitivity. Detection of HBsAg was possible in the concentration range of 10 pg ml^−1^ to 1 µg ml^−1^, with a detection limit of less than 10.0 pg mL^−1^ estimated.

In one study, a protein produced by the fusion of gold binding polypeptides (GBPs) was used to develop an SPR-based sensor for the quick and easy detection of coronavirus [18]. These were immobilized on top of the gold layer and used as a ligand for the surface antigen of SARS-CoV. The proposed sensor worked better when the fusion protein concentration was calibrated to upto 10 µg mL^−1^. The sensor’s detection limit and response time were stated to be 200 ng mL^−1^ within 10 min. SPR was also used to investigate the kinetics of SARS-CoV-2, chimeric SARS-CoV-2, and SARS-CoV receptor binding domains (RBDs) with ACE-2 receptors [199]. Due to the existence of an additional N–O bridge between chimeric SARS-CoV-2 RBD and ACE-2, the study discovered that the chimeric standardized SARS-CoV-2 RBD had a higher binding affinity for ACE-2 receptors. A one-step method was used to prepare DNA–silver nanoparticle (AgNP) conjugates (Figure 5C), which were then used to quantitatively detect HIV DNA using a sandwich strategy based on their powerful SPR scattering signal [200]. This platform could detect HIV DNA in the range of 0.30–2.0 nmol L^−1^. Table 3 presents various SPR-based sensors for virus detection, along with their detection ranges and limits of detection.

### 6.4. Photonic Sensors

The photonic crystals (PCs) are dielectric and nanostructured materials with optical sensing properties that have a wide variety of applications, such as PC-based biosensors, and have been employed to detect various biological molecules and analyte [202,203,204,205]. This is due to the improved sensing performance in terms of sensitivity and selectivity [204]. A label-free biosensor based on inverse opal PC structures for detecting intact Rotavirus was presented in an experimental sample [24]. The peak wavelength value (PWV) shift was used to analyze virus detection success concerning virus concentration. The PWV shift suggested the quantitative concentration of the same target virus agents. The proposed system’s sensitivity was comparable to or better than a commercial ELISA package with a detection limit of 2.54 µg mL^−1^. A photonic biosensor developed with a compact two-dimensional hexagonal PhC over the gold film detected 1.0 × 10^−3^ µg mL^−1^ Epstein–Barr nuclear antigen-1 (EBNA-1) protein [22]. The author also performed the simulation studies, where the simulation results confirm the SPP’s tighter spatial confinement and higher local field strength, which aids in detecting slight changes in the refractive index on the sensor surface. A field diagnostic system based on a combination of advanced bio-sensing and photonics technologies for the detection of swine virus was proposed by Griol et al. [206]. The plan was focused on the use of CMOS (**Complementary Metal Oxide Semiconductor**) compatible microring resonators fabricated in silicon nitride. A detection limit of 7.13 × 10^−6^ RIU was calculated using this planer resonator system.

**Figure 6:**
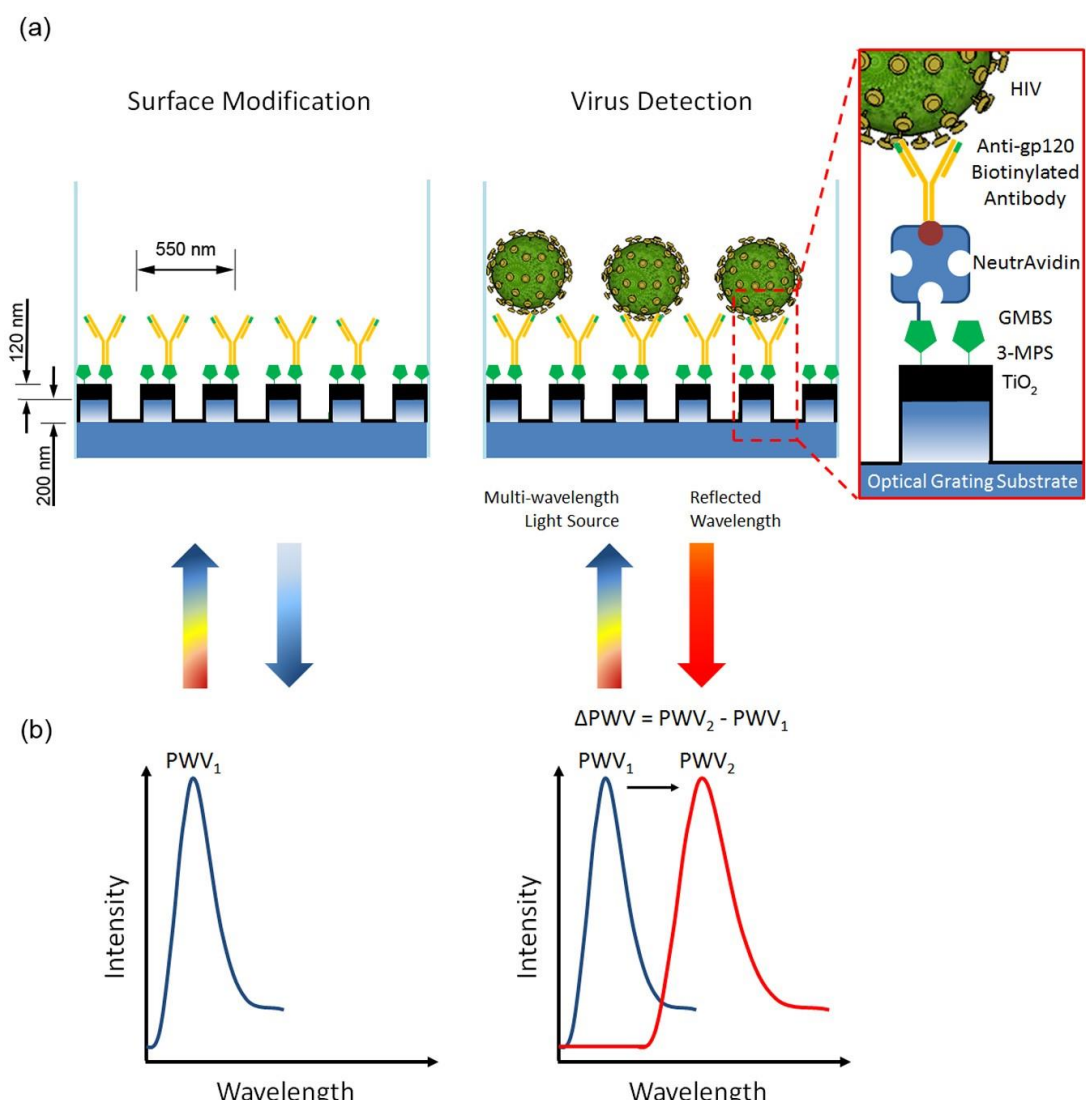
Design and construction of photonic crystal (PC)-based intact virus detection platform. (**a**) The bottom surface of PC biosensor microplate wells comprises a nanostructured subwavelength grating coated with TiO_2_. (**b**) Binding events within the close vicinity of the sensing area change the bulk index of refraction; thus, the reflected light’s peak wavelength value (PWV) is altered. The shift in the peak wavelength (ΔPWV) is directly proportional to the binding of molecules and bioagents (e.g., cells and viruses) onto the biosensing surface (adapted with permission from Shafiee et al., Nature Reports 2014 [65]).

To capture and quantify intact viruses (HIV-1) from biologically relevant samples, a label-free optical sensing mechanism using nanostructured photonic crystals (PC) was developed by Shafiee et al. [65]. During illumination with a broadband light source, the nanostructured surface of the PC biosensor resonantly reflects a narrow wavelength band. Surface-adsorbed bio targets cause a change in the resonant PWV that can be detected with a wavelength resolution of 10 pm, allowing identification of biomolecular layers and a small number of viruses that populate the transducer surface sparsely, as depicted in Figure 6.

The output of a sensitive tapered fiber sensor for label-free detection of DENV II E proteins was developed and investigated. With a sensitivity of 5.02 nm/nM and a detection limit of 1.0 pM, the sensor design successfully detected DENV E proteins [207]. Herein, the researchers have developed and investigated the performance of a sensitive tapered fiber sensor for label-free detection of DENV II E proteins. The designed sensor had enough potential and yielded a sensitivity value of 5.02 nm/nM and a detection limit of 1 pM. Compared to current Dengue diagnostic research efforts, the sensor has achieved higher specificity and sensitivity within a 15-minute detection period. The sensor’s ability to work in experimental conditions was validated by spike and recovery analysis, which showed performance in the range of 83 percent to 89 percent.

In another study by Endo and co-workers, and antibody-immobilized flexible nanoimprint lithography (NIL)-based two-dimensional (2D)-PC biosensor was used to demonstrate the reflectometric detection of influenza virus in human saliva [23]. This resulted in a shift in reflection strength corresponding to influenza virus concentrations, using an antibody-immobilized 2D-PC biosensor. Furthermore, this method detected influenza virions in human saliva (detection limit: 1.0 ng mL^−1^) with a broader working range from 1 pg mL^−1^–100 ng mL^−1^ in a human saliva sample. This method offers a variety of analytical systems to create new rapid diagnostic procedures for severe viral diseases. Table 4 presents various photonics-based sensors for virus detection with their limit of detection and working range.

Very recently, a single-step 15-minute assay capable of detecting as low as 100 pg mL^−1^ human COVID-19 IgG was designed based on photonic resonator absorption microscopy (PRAM) by Zhao et al. [208]. The LOD and LOQ were determined to be 26.7 ± 7.7 and 32.0 ± 8.9 pg mL^−1^, respectively. In this work, for the first time, the Activate Capture + Digital Counting (AC + DC)-based immunoassay was used for rapid and quantitative analysis of serological COVID-19 antibody, showing a path to point-of-care research with a portable detection instrument. This biosensing platform possesses the promising potential to be further extended for multiplexed detection of an analyte of interest.

**Table 4:** Photonic technique-based sensing platform for viral infection.

S. No.	Antigen/Analyte	Bio-Element	Real Sample	Detection Range	LOD	Ref.
1.	Rotavirus	Secondary fluorescent Ab	-	6.35 µg mL^−1^ to 1.27 mg mL^−1^	2.54 µg mL^−1^	[24]
2.	Epstein–Barr virus (EBNA-1) protein	EBNA-1 Antibody	PBS	0.0–10.0 µg mL^−1^	1.0 × 10^−3^ µg mL^−1^	[22]
3.	HIV-1	Antibody	PBS Plasma	10^4^–10^8^ copies mL^−1^ 10^2^–10^7^ copies mL^−1^	-	[65]
4.	DENV E Protein	Antibody	BSA/PBS	1.0 pM–1.0 nM	1.0 pM	[207]
5.	Influenza Virus	Antibody	Human Saliva	1 pg mL^−1^–100 ng mL^−1^	1.0 ng mL^−1^	[23]
6.	SARS-CoV-2	Antibody	Human Serum	-	26.7 ng mL^−1^	[208]
7.	Human Papillomavirus virus-like particles (VLPs)	Antibody	Buffer and Serum (10%)	0.70–5.80 nM	1.5 nM	[209]
8.	Rotavirus	Rotavirus Antibody	Water	10^2^–10^5^ PFU/mL	<10^3^ PFU/mL	[210]

## 7. Conclusions and Future Perspectives

This review highlighted the current trends in optical biosensors and their ability for virus detection such as COVID-19, MERS, SARS, Influenza, Hepatitis, HIV, HPV, Zika, Herpes, and Dengue virus. A brief account of viral molecular structure, disease symptoms, the gold standard of viral diagnostics, and the magnitude of infection caused was mentioned in the introduction section. Later, various biomarkers for viral detection, such as DNA, RNA, peptides, and antibodies, were discussed. An overview of the state of the art for viral diagnostics and sensor development has been discussed. Based on the available literature, the demand for novel biosensors which can match the sensitivity and selectivity of the conventional nucleic acid tests is in high order. Nanomaterial-based biosensors present ideal alternatives due to their advancement in design and fabrication methods, high selectivity, low sensitivity, and good reproducibility. Any biosensor developed for viral disease (COVID-19) and setting for future viral outbreaks must be readily accessible, affordable, and ideally used either as an at-home test or with minimal sample preparation. Optical biosensors provide additional and alternate testing methods that can generate results at a much quicker rate than the tests currently being used for COVID-19 testing.

## Figures and Tables

**Table 1:** Reported colorimetric-based sensing platform for viral infection.

S. No.	Antigen/Analyte	Bio-Element/Detection Strategy	Nanomaterial	Detection/ Linearity Range	LOD	Ref.
1.	DENV	PNA sequences	GNPs	0.00 to 12.0 μM	0.12 μM	[149]
2.	DENV and CHIKV IgG and IgM	Lateral-flow test strip	Red and blue Latex nanoparticles	-	-	[141]
3.	New Caledonia/H1N1/1999 influenza virus	Peroxidase-like activity	AuNP films	10 pg mL^−1^–10 µg mL^−1^	50.50 pg mL^−1^	[156]
4.	Influenza A virus (H3N2)	Peroxidaselike activity	AuNP films	up to 10.0 pg mL^−1^	4.5 pg mL^−1^	[156]
5.	Influenza A virus (H3N2)	M149 antibody (Label-free fluorescence-based detection)	Ab-Polydiacetylene (PDA) Conjugate coated with Polyvinylidene (PVDF) Difluoride Membrane		30 µg mL^−1^	[163]
6.	Influenza H1N1 virus	Anti-influenza A HA-Ab (Ab-E) and HRP-tagged anti-influenza A HA-Ab (HRP-Ab)	Au immunostrip	0 to 10,000 PFU mL^−1^	1.34 PFU mL^−1^ (PBS) 2.27 PFU mL^−1^ (Saliva)	[164]
7.	Avian influenza virus	Fluorescence Reflection	AuNPs and ZnO nanorods	8.0 × 10^5^ to 2.7 × 10^3^ EID_50_/mL	2.7 × 10^4^ EID_50_/mL (naked eye) 8.0 × 10^3^ EID_50_/mL (smartphone device)	[157]
8.	Influenza A virus (H1N1)	Anti-hemagglutinin (HA) mAb	Au Nanozymes and Silica-shelled magnetic nanobeads (MagNBs)	5.0 × 10^−15^–5.0 × 10^−6^ g mL^−1^	5.0 × 10^−12^ g mL^−1^ (human eyes) 44.2 × 10^−15^ g mL^−1^ (microplate reader)	[165]
9.	NPs of influenza A and B virus	STA-RPA70A@biotin-HRP and aptamer	-	0.1 pg mL^−1^ to 1 mg mL^−1^	0.30 pg mL^−1^ 0.16 pg mL^−1^	[166]
10.	Hepatitis B virus	DNA sequences	Cupper nanoclusters	12.0 × 10^9^ to 12.0 × 10^13^ target molecules	12 × 10^9^ molecules	[158]
11.	Hepatitis B virus	HBsAg	Magnetic nanoparticles	0.10–20 ng mL^−1^	0.012 ng mL^−1^	[159]
12.	Hepatitis C virus	HCV oligonucleotides	AuNPs	7.50 × 10^2^ to 2.00 × 10^6^ IU mL^−1^	100 IU mL^−1^ (0.4 IU µL^−1^)	[160]
13.	SARS CoV-2	Colorimetric loop-mediated isothermal amplification (LAMP)	-	625 to 2 × 10^5^ DNA copies	62.5 DNA copies	[161]

LOD: Limit of detection; pg mL^−1^: picogram per mililitre; ng mL^−1^; nanogram per mililitre; µg mL^−1^; microgram per mililitre; ng mL^−1^; µM: micromolar; PFU mL^−1^: plaque forming unit per mililitre; IU mL^−1^: International unit per mililitre.

**Table 2:** Fluorescence/chemiluminescence-based sensing platform for viral infection.

S. No.	Principle	Analyte	Real Sample	Detection Range	LOD	Ref.
1.	**Fluorescence**	Detection of hepatitis B virus DNA (FRET-based)	Human urine sample	0.045–6.0 nmol L^−1^	0.015 nmol L^−1^	[169]
2.		Hepatitis B virus surface antigen (HBsAg) sandwich immunochromatographic assay (ICA)	Human Serum	75.0 pg mL^−1^–4.80 ng mL^−1^ 4.80 pg mL^−1^–75.0 ng mL^−1^	75 pg mL^−1^	[170]
3.		Hepatitis A Virus (HAV)	Human Serum	0.04–6.0 nmol L^−1^	8. 0 nmol L^−1^	[171]
4.		Recombinant hemagglutinin (rHA) protein of the H5N1 influenza virus	Aqueous Buffer Human serum	0.0–200.0 ng mL^−1^	2.0 ng mL^−1^ 3.5 ng mL^−1^	[172]
5.		Human immunodeficiency virus 1 double-stranded DNA sequence Sudan virus RNA sequence	-	-	196 pM 73 pM	[175]
6.		SARS-CoV-2 virus specific genes	TE buffer	0.01–1.0 pM	160 fm	[176]
7.	**Chemiluminescence**	anti-DENV IgM antibodies	Serum	-	-	[177]
8.		Avian influenza viruses (AIV)	Embryonated chicken eggs	1.0 pM–1.0 nM	5.0 pM	[178]
9.		Human T-lymphotropic viruses	Human Serum	1.0 aM–1.0 nM	0.50 aM	[180]
10.		Pseudorabies Virus	Swine	1.0 × 10^1^–1.0 × 10^8^ amol	100 amol	[181]
11.		Hepatitis B surface antigen (HBsAg)	Serum	0.40–15.0 ng mL^−1^	0.30 ng mL^−1^	[182]
12.		HBsAg	Serum	0.12–30 ng mL^−1^	14.0 pg mL^−1^	[183]
13.		H1N1 influenza virus	-	1.0 × 10^−12^–1.0 × 10^−6^ g mL^−1^	1.0 × 10^−13^ g mL^−1^	[189]
14.		Human Immunodeficiency Virus type 1 (HIV-1)	Human Serum	0.020–1.0 pM	5.0 fM	[190]

**Table 3:** Reported SPR-based sensing platform for viral infection.

S. No.	Antigen/Analyte	Bio-Element	Real Sample	Detection Range	LOD	Ref.
1.	Avian Influenza Virus H5N1	Aptamer	Poultry swab	0.128 to 1.28 HAU	0.128 HAU	[193]
2.	Avian influenza virus subtype H6	Monoclonal antibodies	Tracheal samples from chicken	0.50–10 µg mL^−1^	5.14 × 10^5^ EID_50_/0.1 mL	[158]
3.	Influenza Virus	Antibodies/Protein	Serum	0.50–10 µg mL^−1^	<0.50 µg mL^−1^	[194]
4.	DENV protein	IgM Ab	-	0.08 pM to 0.5 pM	0.08 pM	[197]
5.	Human hepatitis virus surface antigen (HBsAg)	Antibodies	-	10 pg mL^−1^–1 µg mL^−1^	10 pg mL^−1^	[198]
6.	SARS-CoV	Goldbinding polypeptides (GBPs)	-	0–10 µg mL^−1^	200 ng mL^−1^	[18]
7.	Hepatitis B	Monoclonal hepatitis B (HBsAb)	buffer, blood serum and plasma	0.01–1.0 IU/mL	0.01 IU/mL	[201]
8.	HIV DNA	DNA	-	0.30–2.0 nmol L^−1^	~195 pmol L^−1^	[200]

## Data Availability

Not applicable.
